# Bis{1,2-bis­[bis­(3-hydroxy­prop­yl)phosphino]ethane}dichloridoiron(II)

**DOI:** 10.1107/S160053681001768X

**Published:** 2010-05-22

**Authors:** Justin L. Crossland, Lev N. Zakharov, David R. Tyler

**Affiliations:** a Department of Chemistry, 1253 University of Oregon, Eugene, Oregon 97403-1253, USA

## Abstract

In the title compound, [FeCl_2_(C_14_H_32_O_4_P_2_)_2_], the Fe^II^ atom (site symmetry 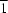) adopts a distorted *trans*-FeCl_2_P_4_ octa­hedral geometry with two *P*,*P*′-bidentate ligands in the equatorial positions and two chloride ions in the axial positions. In the crystal, mol­ecules are linked by O—H⋯O and O—H⋯Cl hydrogen bonds, generating a three-dimensional network.

## Related literature

For background to the applications of iron–diphosphine complexes, see: Lyon (1993 ▶); Miller *et al.* (2002 ▶). For further synthetic details, see: Baxley *et al.* (1996 ▶).
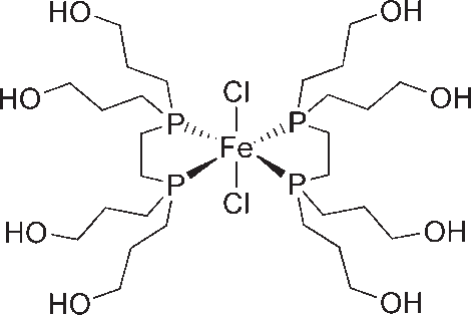


## Experimental

### 

#### Crystal data

[FeCl_2_(C_14_H_32_O_4_P_2_)_2_]
*M* *_r_* = 779.42
Triclinic, 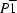

*a* = 8.7120 (4) Å
*b* = 10.4252 (5) Å
*c* = 10.7441 (5) Å
α = 96.086 (1)°
β = 104.215 (1)°
γ = 105.860 (1)°
*V* = 894.12 (7) Å^3^
*Z* = 1
Mo *K*α radiation
μ = 0.80 mm^−1^
*T* = 173 K
0.29 × 0.26 × 0.18 mm

#### Data collection

Bruker APEX CCD diffractometer
Absorption correction: multi-scan (*SADABS*; Bruker, 2000 ▶) *T*_min_ = 0.802, *T*_max_ = 0.870
10053 measured reflections
3863 independent reflections
3693 reflections with *I* > 2σ(*I*)
*R*_int_ = 0.015

#### Refinement

*R*[*F* ^2^ > 2σ(*F* ^2^)] = 0.026
*wR*(*F* ^2^) = 0.068
*S* = 1.06
3863 reflections
212 parameters
H atoms treated by a mixture of independent and constrained refinement
Δρ_max_ = 0.47 e Å^−3^
Δρ_min_ = −0.47 e Å^−3^

### 

Data collection: *SMART* (Bruker, 2000 ▶); cell refinement: *SAINT* (Bruker, 2000 ▶); data reduction: *SAINT*; program(s) used to solve structure: *SHELXTL* (Sheldrick, 2008 ▶); program(s) used to refine structure: *SHELXTL*; molecular graphics: *SHELXTL*; software used to prepare material for publication: *SHELXTL*.

## Supplementary Material

Crystal structure: contains datablocks I, global. DOI: 10.1107/S160053681001768X/hb5437sup1.cif

Structure factors: contains datablocks I. DOI: 10.1107/S160053681001768X/hb5437Isup2.hkl

Additional supplementary materials:  crystallographic information; 3D view; checkCIF report

## Figures and Tables

**Table:** 

Fe1—P1	2.2790 (3)
Fe1—P2	2.3008 (3)
Fe1—Cl1	2.3507 (3)

**Table:** 

P1—Fe1—P2	85.008 (12)

**Table 2:** Hydrogen-bond geometry (Å, °)

*D*—H⋯*A*	*D*—H	H⋯*A*	*D*⋯*A*	*D*—H⋯*A*
O1—H1*O*⋯O3^i^	0.79 (3)	1.98 (3)	2.7660 (18)	174 (3)
O2—H2*O*⋯O1^ii^	0.73 (2)	2.12 (2)	2.8548 (17)	174 (2)
O3—H3*O*⋯O4^iii^	0.81 (3)	1.93 (3)	2.7370 (19)	171 (3)
O4—H4*O*⋯Cl1^iv^	0.84 (3)	2.28 (3)	3.1150 (13)	171 (3)

Symmetry codes: (i) 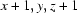; (ii) 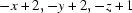; (iii) 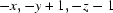; (iv) 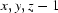.

**Fig. 1.**
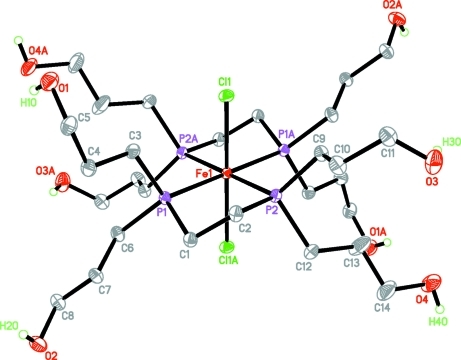
The structure of (I) with 50% probability displacement ellipsoids (H atoms are omitted for clarity). Symmetry code: (i): -x+1, -y+1, -z.

## supplementary crystallographic information

### Comment

Iron-diphosphine complexes containing water-soluble phosphine ligands have shown promise as dinitrogen scrubbers for nitrogen-containing natural gas streams (Lyon, 1993). Sulfonated phosphines have been used to impart water-solubility, however the sulfonate groups are often non-innocent and can prevent iron complexes from binding N_2_. We have focused on hydroxyl functionalized phosphine ligands, such as DHPrPE (DHPrPE = 1,2-bis(dihydroxypropylphosphino)ethane) to synthesize water-soluble iron complexes capable of binding dinitrogen (Miller *et al.*, 2002). However, using this particular ligand we were previously unable to isolate the *trans* dichloride complex, which is the required isomer to achieve dinitrogen binding. Here we report the synthesis and structural characterization of *trans*-Fe(DHPrPE)_2_Cl_2_ (DHPrPE = 1,2-bis(dihydroxypropylphosphino)ethane).

The structure of Fe[(CH_2_CH_2_)P_2_(CH_2_CH_2_CH_2_OH)_2_]_2_Cl_2_ is centrosymmetyrical. The Fe atom has a distorted octahedral coordination with four P atoms in equatorial positions and two Cl atoms in apical positions (Fig. 1). The Fe(1)—Cl distance is 2.3507 (3) Å, the Fe(1)—P(1,2) distances are 2.2790 (3) and 2.3008 (3) Å, respectively. All -OH groups are involved in intra- and inter-molecular O—H···Cl and O—H···O H-bonds (Table 1).

### Experimental

1,2-bis(dihydroxypropylphosphino)ethane (DHPrPE) was synthesized as previously reported (Baxley *et al.*, 1996). *trans*-Fe(DHPrPE)_2_Cl_2_ was prepared by adding DHPrPE (0.33 g, 1.01 mmol) to a stirring solution of FeCl_2_(H_2_O)_4_ (0.10 g, 0.505 mmol) in 20 ml of methanol, giving a deep purple solution. The ^31^P{^1^H} NMR spectrum of the purple solution at 233 K showed three resonances (79, 71, and 53 ppm), likely due to a mixture of *trans* and *cis* isomers. The purple solution was layered with diethyl ether and allowed to stand at room temperature for one week. After this time a few lime green blocks of (I) were isolated from the purple mother liquor. The ^31^P{^1^H} NMR spectrum of the green crystals at 233 K showed a single resonance at 53 ppm.

### Refinement

The H atoms in CH_2_ groups were positioned geometrically and refined in the riding model approximation, C—H = 0.99 Å; U_iso_(H) = 1.2U_eq_(C). The H atoms in -OH groups were found from the residual density map and refined with isotropic thermal parameters. There are eight flexible (CH_2_CH_2_CH_2_OH) groups in the structure and as a result there are elongations of displacement ellipsoids for some atoms.

### Crystal data

**Table:**

[FeCl_2_(C_14_H_32_O_4_P_2_)_2_]	*Z* = 1
*M**_r_* = 779.42	*F*(000) = 416
Triclinic, *P*1	*D*_x_ = 1.448 Mg m^−^^3^
Hall symbol: -P 1	Mo *K*α radiation, λ = 0.71073 Å
*a* = 8.7120 (4) Å	Cell parameters from 7234 reflections
*b* = 10.4252 (5) Å	θ = 2.5–28.3°
*c* = 10.7441 (5) Å	µ = 0.80 mm^−^^1^
α = 96.086 (1)°	*T* = 173 K
β = 104.215 (1)°	Block, lime green
γ = 105.860 (1)°	0.29 × 0.26 × 0.18 mm
*V* = 894.12 (7) Å^3^	

### Data collection

**Table:**

Bruker APEX CCD diffractometer	3863 independent reflections
Radiation source: fine-focus sealed tube	3693 reflections with *I* > 2σ(*I*)
graphite	*R*_int_ = 0.015
phi and ω scans	θ_max_ = 27.0°, θ_min_ = 2.0°
Absorption correction: multi-scan (*SADABS*; Bruker, 2000)	*h* = −11→11
*T*_min_ = 0.802, *T*_max_ = 0.870	*k* = −13→13
10053 measured reflections	*l* = −13→13

### Refinement

**Table:**

Refinement on *F*^2^	Primary atom site location: structure-invariant direct methods
Least-squares matrix: full	Secondary atom site location: difference Fourier map
*R*[*F*^2^ > 2σ(*F*^2^)] = 0.026	Hydrogen site location: inferred from neighbouring sites
*wR*(*F*^2^) = 0.068	H atoms treated by a mixture of independent and constrained refinement
*S* = 1.06	*w* = 1/[σ^2^(*F*_o_^2^) + (0.0355*P*)^2^ + 0.5361*P*] where *P* = (*F*_o_^2^ + 2*F*_c_^2^)/3
3863 reflections	(Δ/σ)_max_ = 0.001
212 parameters	Δρ_max_ = 0.47 e Å^−^^3^
0 restraints	Δρ_min_ = −0.47 e Å^−^^3^

### Special details

**Table:**

Geometry. All esds (except the esd in the dihedral angle between two l.s. planes) are estimated using the full covariance matrix. The cell esds are taken into account individually in the estimation of esds in distances, angles and torsion angles; correlations between esds in cell parameters are only used when they are defined by crystal symmetry. An approximate (isotropic) treatment of cell esds is used for estimating esds involving l.s. planes.
Refinement. Refinement of *F*^2^ against ALL reflections. The weighted *R*-factor wR and goodness of fit *S* are based on *F*^2^, conventional *R*-factors *R* are based on *F*, with *F* set to zero for negative *F*^2^. The threshold expression of *F*^2^ > σ(*F*^2^) is used only for calculating *R*-factors(gt) etc. and is not relevant to the choice of reflections for refinement. *R*-factors based on *F*^2^ are statistically about twice as large as those based on *F*, and *R*- factors based on ALL data will be even larger.

### Fractional atomic coordinates and isotropic or equivalent isotropic displacement parameters (Å^2^)

**Table:**

	*x*	*y*	*z*	*U*_iso_*/*U*_eq_	
Fe1	0.5000	0.5000	0.0000	0.01036 (8)	
Cl1	0.31246 (4)	0.50199 (3)	0.12398 (3)	0.01664 (9)	
P1	0.69987 (4)	0.68781 (3)	0.13091 (3)	0.01216 (9)	
P2	0.40130 (4)	0.65190 (3)	−0.11033 (3)	0.01239 (9)	
O1	0.68912 (15)	0.87217 (12)	0.56263 (11)	0.0248 (2)	
O2	1.34336 (14)	0.95555 (12)	0.22692 (12)	0.0234 (2)	
O3	−0.07362 (16)	0.74720 (14)	−0.34828 (12)	0.0309 (3)	
O4	0.19987 (17)	0.52861 (13)	−0.62339 (12)	0.0335 (3)	
C1	0.70197 (17)	0.82669 (14)	0.03860 (14)	0.0154 (3)	
H1A	0.7685	0.9149	0.0967	0.019*	
H1B	0.7522	0.8142	−0.0332	0.019*	
C2	0.52256 (18)	0.82418 (14)	−0.01648 (14)	0.0158 (3)	
H2A	0.5174	0.8919	−0.0741	0.019*	
H2B	0.4763	0.8465	0.0554	0.019*	
C3	0.65904 (18)	0.75708 (14)	0.28043 (14)	0.0168 (3)	
H3A	0.5491	0.7722	0.2545	0.020*	
H3B	0.6497	0.6867	0.3356	0.020*	
C4	0.78515 (19)	0.88863 (15)	0.36498 (15)	0.0206 (3)	
H4A	0.8880	0.8695	0.4096	0.025*	
H4B	0.8144	0.9541	0.3078	0.025*	
C5	0.7216 (2)	0.95323 (15)	0.46720 (15)	0.0213 (3)	
H5A	0.8050	1.0417	0.5123	0.026*	
H5B	0.6177	0.9709	0.4225	0.026*	
C6	0.92139 (17)	0.69512 (14)	0.18114 (14)	0.0166 (3)	
H6A	0.9467	0.6712	0.2690	0.020*	
H6B	0.9368	0.6247	0.1202	0.020*	
C7	1.04890 (17)	0.83151 (14)	0.18558 (14)	0.0168 (3)	
H7A	1.0265	0.8555	0.0976	0.020*	
H7B	1.0349	0.9028	0.2464	0.020*	
C8	1.22756 (18)	0.82938 (15)	0.22905 (15)	0.0192 (3)	
H8A	1.2522	0.8099	0.3188	0.023*	
H8B	1.2409	0.7557	0.1706	0.023*	
C9	0.18524 (17)	0.65570 (14)	−0.13785 (14)	0.0160 (3)	
H9A	0.1138	0.5890	−0.2176	0.019*	
H9B	0.1454	0.6270	−0.0634	0.019*	
C10	0.16433 (19)	0.79529 (15)	−0.15321 (16)	0.0204 (3)	
H10A	0.2110	0.8562	−0.0667	0.024*	
H10B	0.2292	0.8347	−0.2114	0.024*	
C11	−0.01607 (19)	0.78978 (15)	−0.20895 (16)	0.0215 (3)	
H11A	−0.0872	0.7260	−0.1688	0.026*	
H11B	−0.0269	0.8808	−0.1860	0.026*	
C12	0.43480 (19)	0.67282 (16)	−0.27098 (14)	0.0197 (3)	
H12A	0.4471	0.7684	−0.2800	0.024*	
H12B	0.5416	0.6569	−0.2715	0.024*	
C13	0.3005 (3)	0.58188 (19)	−0.39026 (16)	0.0370 (5)	
H13A	0.2926	0.4861	−0.3855	0.044*	
H13B	0.1920	0.5936	−0.3886	0.044*	
C14	0.3314 (3)	0.6099 (2)	−0.51359 (17)	0.0419 (5)	
H14A	0.3452	0.7069	−0.5168	0.050*	
H14B	0.4365	0.5930	−0.5180	0.050*	
H1O	0.758 (3)	0.837 (2)	0.583 (2)	0.045 (7)*	
H2O	1.337 (3)	1.004 (2)	0.279 (2)	0.031 (6)*	
H3O	−0.109 (3)	0.665 (3)	−0.364 (3)	0.053 (8)*	
H4O	0.238 (3)	0.531 (3)	−0.688 (3)	0.059 (8)*	

### Atomic displacement parameters (Å^2^)

**Table:**

	*U*^11^	*U*^22^	*U*^33^	*U*^12^	*U*^13^	*U*^23^
Fe1	0.01193 (14)	0.00882 (13)	0.00954 (13)	0.00263 (10)	0.00279 (10)	0.00068 (10)
Cl1	0.01817 (17)	0.01714 (17)	0.01571 (17)	0.00498 (13)	0.00786 (13)	0.00194 (12)
P1	0.01309 (17)	0.00982 (16)	0.01179 (17)	0.00240 (13)	0.00236 (13)	0.00038 (12)
P2	0.01403 (17)	0.01074 (17)	0.01157 (17)	0.00349 (13)	0.00256 (13)	0.00212 (13)
O1	0.0274 (6)	0.0288 (6)	0.0194 (6)	0.0108 (5)	0.0071 (5)	0.0024 (5)
O2	0.0183 (5)	0.0214 (6)	0.0274 (6)	0.0007 (4)	0.0093 (5)	−0.0008 (5)
O3	0.0328 (7)	0.0297 (7)	0.0267 (6)	0.0113 (5)	−0.0006 (5)	0.0082 (5)
O4	0.0410 (7)	0.0356 (7)	0.0143 (6)	−0.0014 (6)	0.0085 (5)	−0.0019 (5)
C1	0.0158 (7)	0.0116 (6)	0.0159 (7)	0.0016 (5)	0.0022 (5)	0.0025 (5)
C2	0.0178 (7)	0.0111 (6)	0.0163 (7)	0.0037 (5)	0.0022 (5)	0.0018 (5)
C3	0.0192 (7)	0.0155 (7)	0.0136 (6)	0.0034 (5)	0.0048 (5)	−0.0009 (5)
C4	0.0207 (7)	0.0189 (7)	0.0173 (7)	0.0020 (6)	0.0045 (6)	−0.0038 (6)
C5	0.0256 (8)	0.0191 (7)	0.0166 (7)	0.0064 (6)	0.0042 (6)	−0.0024 (6)
C6	0.0146 (7)	0.0135 (7)	0.0197 (7)	0.0040 (5)	0.0023 (5)	0.0017 (5)
C7	0.0149 (7)	0.0148 (7)	0.0192 (7)	0.0037 (5)	0.0037 (5)	0.0023 (5)
C8	0.0152 (7)	0.0175 (7)	0.0223 (7)	0.0038 (5)	0.0039 (6)	0.0002 (6)
C9	0.0155 (7)	0.0134 (6)	0.0183 (7)	0.0050 (5)	0.0029 (5)	0.0033 (5)
C10	0.0186 (7)	0.0146 (7)	0.0275 (8)	0.0063 (6)	0.0040 (6)	0.0057 (6)
C11	0.0205 (7)	0.0176 (7)	0.0275 (8)	0.0087 (6)	0.0053 (6)	0.0053 (6)
C12	0.0220 (7)	0.0227 (7)	0.0154 (7)	0.0067 (6)	0.0057 (6)	0.0075 (6)
C13	0.0521 (12)	0.0281 (9)	0.0166 (8)	−0.0088 (8)	0.0096 (8)	0.0009 (7)
C14	0.0450 (11)	0.0479 (12)	0.0166 (8)	−0.0061 (9)	0.0030 (8)	0.0052 (8)

### Geometric parameters (Å, °)

**Table:**

Fe1—P1^i^	2.2790 (3)	C4—H4A	0.9900
Fe1—P1	2.2790 (3)	C4—H4B	0.9900
Fe1—P2	2.3008 (3)	C5—H5A	0.9900
Fe1—P2^i^	2.3008 (3)	C5—H5B	0.9900
Fe1—Cl1^i^	2.3507 (3)	C6—C7	1.5344 (19)
Fe1—Cl1	2.3507 (3)	C6—H6A	0.9900
P1—C1	1.8383 (14)	C6—H6B	0.9900
P1—C3	1.8449 (14)	C7—C8	1.518 (2)
P1—C6	1.8496 (14)	C7—H7A	0.9900
P2—C9	1.8445 (14)	C7—H7B	0.9900
P2—C12	1.8452 (15)	C8—H8A	0.9900
P2—C2	1.8478 (14)	C8—H8B	0.9900
O1—C5	1.4287 (19)	C9—C10	1.5360 (19)
O1—H1O	0.79 (3)	C9—H9A	0.9900
O2—C8	1.4285 (18)	C9—H9B	0.9900
O2—H2O	0.73 (2)	C10—C11	1.521 (2)
O3—C11	1.435 (2)	C10—H10A	0.9900
O3—H3O	0.81 (3)	C10—H10B	0.9900
O4—C14	1.420 (2)	C11—H11A	0.9900
O4—H4O	0.84 (3)	C11—H11B	0.9900
C1—C2	1.5212 (19)	C12—C13	1.520 (2)
C1—H1A	0.9900	C12—H12A	0.9900
C1—H1B	0.9900	C12—H12B	0.9900
C2—H2A	0.9900	C13—C14	1.459 (2)
C2—H2B	0.9900	C13—H13A	0.9900
C3—C4	1.528 (2)	C13—H13B	0.9900
C3—H3A	0.9900	C14—H14A	0.9900
C3—H3B	0.9900	C14—H14B	0.9900
C4—C5	1.521 (2)		
			
P1^i^—Fe1—P1	180.0	O1—C5—H5B	108.8
P1^i^—Fe1—P2	94.992 (12)	C4—C5—H5B	108.8
P1—Fe1—P2	85.008 (12)	H5A—C5—H5B	107.7
P1^i^—Fe1—P2^i^	85.008 (12)	C7—C6—P1	116.21 (10)
P1—Fe1—P2^i^	94.992 (12)	C7—C6—H6A	108.2
P2—Fe1—P2^i^	180.0	P1—C6—H6A	108.2
P1^i^—Fe1—Cl1^i^	93.761 (12)	C7—C6—H6B	108.2
P1—Fe1—Cl1^i^	86.239 (12)	P1—C6—H6B	108.2
P2—Fe1—Cl1^i^	91.646 (12)	H6A—C6—H6B	107.4
P2^i^—Fe1—Cl1^i^	88.354 (12)	C8—C7—C6	112.88 (12)
P1^i^—Fe1—Cl1	86.239 (12)	C8—C7—H7A	109.0
P1—Fe1—Cl1	93.761 (12)	C6—C7—H7A	109.0
P2—Fe1—Cl1	88.354 (12)	C8—C7—H7B	109.0
P2^i^—Fe1—Cl1	91.646 (12)	C6—C7—H7B	109.0
Cl1^i^—Fe1—Cl1	180.0	H7A—C7—H7B	107.8
C1—P1—C3	101.59 (7)	O2—C8—C7	111.79 (12)
C1—P1—C6	102.93 (7)	O2—C8—H8A	109.3
C3—P1—C6	105.40 (7)	C7—C8—H8A	109.3
C1—P1—Fe1	106.37 (5)	O2—C8—H8B	109.3
C3—P1—Fe1	116.80 (5)	C7—C8—H8B	109.3
C6—P1—Fe1	121.13 (5)	H8A—C8—H8B	107.9
C9—P2—C12	101.59 (7)	C10—C9—P2	114.10 (10)
C9—P2—C2	101.99 (6)	C10—C9—H9A	108.7
C12—P2—C2	99.55 (7)	P2—C9—H9A	108.7
C9—P2—Fe1	122.82 (5)	C10—C9—H9B	108.7
C12—P2—Fe1	119.70 (5)	P2—C9—H9B	108.7
C2—P2—Fe1	107.56 (5)	H9A—C9—H9B	107.6
C5—O1—H1O	110.5 (18)	C11—C10—C9	113.31 (12)
C8—O2—H2O	104.6 (17)	C11—C10—H10A	108.9
C11—O3—H3O	107.7 (19)	C9—C10—H10A	108.9
C14—O4—H4O	106.7 (19)	C11—C10—H10B	108.9
C2—C1—P1	107.68 (9)	C9—C10—H10B	108.9
C2—C1—H1A	110.2	H10A—C10—H10B	107.7
P1—C1—H1A	110.2	O3—C11—C10	112.15 (13)
C2—C1—H1B	110.2	O3—C11—H11A	109.2
P1—C1—H1B	110.2	C10—C11—H11A	109.2
H1A—C1—H1B	108.5	O3—C11—H11B	109.2
C1—C2—P2	107.90 (9)	C10—C11—H11B	109.2
C1—C2—H2A	110.1	H11A—C11—H11B	107.9
P2—C2—H2A	110.1	C13—C12—P2	116.73 (11)
C1—C2—H2B	110.1	C13—C12—H12A	108.1
P2—C2—H2B	110.1	P2—C12—H12A	108.1
H2A—C2—H2B	108.4	C13—C12—H12B	108.1
C4—C3—P1	117.88 (10)	P2—C12—H12B	108.1
C4—C3—H3A	107.8	H12A—C12—H12B	107.3
P1—C3—H3A	107.8	C14—C13—C12	113.40 (15)
C4—C3—H3B	107.8	C14—C13—H13A	108.9
P1—C3—H3B	107.8	C12—C13—H13A	108.9
H3A—C3—H3B	107.2	C14—C13—H13B	108.9
C5—C4—C3	113.34 (13)	C12—C13—H13B	108.9
C5—C4—H4A	108.9	H13A—C13—H13B	107.7
C3—C4—H4A	108.9	O4—C14—C13	112.24 (16)
C5—C4—H4B	108.9	O4—C14—H14A	109.2
C3—C4—H4B	108.9	C13—C14—H14A	109.2
H4A—C4—H4B	107.7	O4—C14—H14B	109.2
O1—C5—C4	113.85 (13)	C13—C14—H14B	109.2
O1—C5—H5A	108.8	H14A—C14—H14B	107.9
C4—C5—H5A	108.8		
			
P2—Fe1—P1—C1	−18.86 (5)	C6—P1—C1—C2	176.02 (9)
P2^i^—Fe1—P1—C1	161.14 (5)	Fe1—P1—C1—C2	47.68 (10)
Cl1^i^—Fe1—P1—C1	73.13 (5)	P1—C1—C2—P2	−54.31 (11)
Cl1—Fe1—P1—C1	−106.87 (5)	C9—P2—C2—C1	168.04 (10)
P2—Fe1—P1—C3	93.66 (5)	C12—P2—C2—C1	−87.83 (10)
P2^i^—Fe1—P1—C3	−86.34 (5)	Fe1—P2—C2—C1	37.63 (10)
Cl1^i^—Fe1—P1—C3	−174.36 (5)	C1—P1—C3—C4	−60.57 (12)
Cl1—Fe1—P1—C3	5.64 (5)	C6—P1—C3—C4	46.50 (13)
P1^i^—Fe1—P1—C6	117 (100)	Fe1—P1—C3—C4	−175.77 (9)
P2—Fe1—P1—C6	−135.60 (6)	P1—C3—C4—C5	166.97 (11)
P2^i^—Fe1—P1—C6	44.40 (6)	C3—C4—C5—O1	63.80 (17)
Cl1^i^—Fe1—P1—C6	−43.61 (6)	C1—P1—C6—C7	22.64 (12)
Cl1—Fe1—P1—C6	136.39 (6)	C3—P1—C6—C7	−83.45 (12)
P1^i^—Fe1—P2—C9	55.49 (6)	Fe1—P1—C6—C7	141.10 (9)
P1—Fe1—P2—C9	−124.51 (6)	P1—C6—C7—C8	179.38 (10)
Cl1^i^—Fe1—P2—C9	149.41 (6)	C6—C7—C8—O2	177.46 (12)
Cl1—Fe1—P2—C9	−30.59 (6)	C12—P2—C9—C10	−69.66 (12)
P1^i^—Fe1—P2—C12	−74.53 (6)	C2—P2—C9—C10	32.86 (12)
P1—Fe1—P2—C12	105.47 (6)	Fe1—P2—C9—C10	153.12 (9)
Cl1^i^—Fe1—P2—C12	19.39 (6)	P2—C9—C10—C11	165.53 (11)
Cl1—Fe1—P2—C12	−160.61 (6)	C9—C10—C11—O3	−80.07 (16)
P1^i^—Fe1—P2—C2	173.09 (5)	C9—P2—C12—C13	−48.66 (15)
P1—Fe1—P2—C2	−6.91 (5)	C2—P2—C12—C13	−153.12 (14)
Cl1^i^—Fe1—P2—C2	−92.99 (5)	Fe1—P2—C12—C13	90.26 (14)
Cl1—Fe1—P2—C2	87.01 (5)	P2—C12—C13—C14	176.65 (16)
C3—P1—C1—C2	−74.99 (10)	C12—C13—C14—O4	−176.86 (17)

Symmetry codes: (i) −*x*+1, −*y*+1, −*z*.

### Hydrogen-bond geometry (Å, °)

**Table:**

*D*—H···*A*	*D*—H	H···*A*	*D*···*A*	*D*—H···*A*
O1—H1O···O3^ii^	0.79 (3)	1.98 (3)	2.7660 (18)	174 (3)
O2—H2O···O1^iii^	0.73 (2)	2.12 (2)	2.8548 (17)	174 (2)
O3—H3O···O4^iv^	0.81 (3)	1.93 (3)	2.7370 (19)	171 (3)
O4—H4O···Cl1^v^	0.84 (3)	2.28 (3)	3.1150 (13)	171 (3)

Symmetry codes: (ii) *x*+1, *y*, *z*+1; (iii) −*x*+2, −*y*+2, −*z*+1; (iv) −*x*, −*y*+1, −*z*−1; (v) *x*, *y*, *z*−1.
